# Granulomatous interstitial nephritis due to chronic lymphocytic leukemia: a case report

**DOI:** 10.1186/s12882-017-0775-3

**Published:** 2017-12-02

**Authors:** Yasuo Suzuki, Kan Katayama, Eiji Ishikawa, Shoko Mizoguchi, Keiko Oda, Yosuke Hirabayashi, Ayumi Haruki, Takayasu Ito, Mika Fujimoto, Tomohiro Murata, Masaaki Ito

**Affiliations:** Department of Cardiology and Nephrology, Mie University Graduate School of Medicine, 2-174 Edobashi, Tsu, Mie, 514-8507 Japan

**Keywords:** Chronic lymphocytic leukemia, Epithelioid cell granuloma, Interstitial nephritis

## Abstract

**Background:**

Renal failure due to the infiltration of chronic lymphocytic leukemia (CLL) cells into the tubulointerstitial area of the kidney is uncommon. Furthermore, granulomatous interstitial nephritis (GIN) is a rare histological diagnosis in patients undergoing a renal biopsy. We herein report a case of GIN due to the diffuse infiltration of CLL cells in a patient who developed progressive renal failure.

**Case presentation:**

The patient was a 55-year-old man who had been diagnosed with CLL 4 years earlier and who had been followed up without treatment. Although his serum creatinine level had remained normal for three and a half years, it started to increase in the six months prior to his presentation. A urinalysis showed mild proteinuria without any hematuria at the time of presentation. A renal biopsy revealed the diffuse infiltration of CLL cells into the tubulointerstitial area with non-caseating epithelioid cell granulomas. Despite cyclophosphamide treatment, his renal function did not improve, and he ultimately required maintenance hemodialysis.

**Conclusion:**

When progressive renal failure is combined with CLL, GIN due to the direct infiltration of CLL cells should be considered as a differential diagnosis.

## Background

Chronic lymphocytic leukemia (CLL) mainly invades the lymph nodes, bone marrow, liver, and spleen; extramedullary lesions of CLL are uncommon in the clinical setting. Although extramedullary lesions of CLL are observed on the skin and in the central nervous system of 33% and 27% of CLL patients, respectively, genitourinary/gynecological lesions are only seen in approximately 10% of CLL patients [1]. In contrast, kidney infiltration is found on autopsy in 63%–90% of CLL patients [2–4]; the lesions are nodular or diffuse in the subcapsular cortex, cortico-medullary junction or along the vasa recta [3]. However, these lesions seldom lead to end-stage renal failure [5].

Furthermore, granulomatous interstitial nephritis (GIN) is a rare histological diagnosis, being detected in only 0.5%–5.9% of renal biopsy cases [6, 7]. We herein report a case of GIN due to the infiltration of CLL cells into the tubulointerstitial area.

## Case presentation

The patient was a 55-year-old man in whom leukocytosis and systemic enlarged lymph nodes had been pointed out on positron emission tomography-computed tomography (PET-CT), which had been performed in an annual health check four years previously. The urinalysis findings at that time were normal without hematuria or proteinuria. He was referred to a hematologist in a regional hospital. Peripheral blood flow cytometry revealed that the percentages of lymphocytes that were positive for cluster of differentiation (CD)5, CD19, CD20, and CD23 to be 97.5%, 91.7%, 80.7%, and 84.3%, respectively. He was diagnosed with CLL Rai stage II. At the time, his serum creatinine level was 0.69 mg/dl and he was not taking any medications, including traditional Chinese medicines. He was followed up without treatment, and his serum creatinine level remained normal for three and a half years. However, his serum creatinine level gradually increased from 1.11 to 2.66 mg/dL in the 6 months before he presented to our hospital. He was referred to our hospital to undergo an evaluation for progressive renal dysfunction.

On admission, his height was 167 cm, and his body weight was 70 kg. His body temperature was 36.8 °C, and his heart rate was 60 beats per minute. His blood pressure was 108/69 mmHg. A physical examination revealed the palpable enlargement of the cervical and inguinal lymph nodes, liver, and spleen. The laboratory data on admission are summarized in Table 1. The patient’s urinary protein level was 0.23 g per day without any occult blood. The levels of urinary N-acetyl-β-D-glucosaminidase and β_2_ microglobulin were high. Leukocytosis was observed (91.8% lymphocytes). The blood urea nitrogen and serum creatinine levels were 40 mg/dL and 3.89 mg/dL, respectively. The patient was negative for myeloperoxidase antineutrophil cytoplasmic antibody, proteinase3-antineutrophil cytoplasmic antibody, M-protein, and Bence Jones protein. The patient was also showed negative results for the interferon gamma release assay (IGRA). The patient’s angiotensin-converting enzyme (ACE) level was normal. No bilateral hilar adenopathy or infiltration was detected on a chest radiograph. No uveitis was detected in an ophthalmic examination. Abdominal echography showed that the right and left kidneys were 10.7 × 4.8 cm and 10.6 × 5.3 cm in size, respectively.

**Table 1:** The laboratory data on admission

Hematology		Serology	
WBC	108,750 /μL	CRP	0.84 mg/dL
Neut	8%	PR3-ANCA	(−)
Lymp	91.8%	MPO-ANCA	(−)
RBC	351 × 10^4^ /μL	ACE	10.4 U/L
Hb	10.3 g/dL	IFE	Monoclonal protein (−)
Ht	32.1%	IGRA	(−)
Plt	20.5 × 10^4^ /μL		
Blood chemistry		Coagulation	
TP	6.6 g/dL	APTT	28.6 sec
Alb	4.5 g/dL	PT	10.8 sec
BUN	40 mg/dL	PT-INR	1.02
Cr	3.89 mg/dL		
LDH	155 IU/L	Urinalysis	
UA	7.0 mg/dL	Occult blood	(−)
Na	139 mEq/L	Protein	0.23 g/day
K	4.3 mEq/L	β_2_mg	44,341 μg/L
Cl	99 mEq/L	NAG	31.7 U/L
Ca	9.1 mg/dL	BJP	(−)
IP	3.6 mg/dL		
HCO_3_^−^	23.8 mEq/L		

*WBC* white blood cells, *Neut* neutrophil, *Lymp* lymphocyte, *RBC* red blood cells, *Hb*, hemoglobin, *Ht* hematocrit, *TP* total protein, *Alb* albumin, *BUN* blood urea nitrogen, *Cr* creatinine, *LDH* lactic dehydrogenation enzyme, *UA* uric acid, *Na* natrium, *K* kalium, *Cl* chloride, *Ca* calcium, *IP* inorganic phosphates, *HCO*_*3*_^*−*^ bicarbonate ion, *CRP* C-reactive protein, *PR3-ANCA* proteinase3-antineutrophil cytoplasmic antibody, *MPO-ANCA* myeloperoxidase antineutrophil cytoplasmic antibody, *ACE* angiotensin converting enzyme, *IFE* immunofixation electrophoresis, *IGRA* Interferon Gamma Release Assay, *APTT* activated partial thromboplastin time, *PT* prothrombin time, *PT-INR* prothrombin time international normalized ratio, *β*_*2*_ *mg* β_2_microglobulin, *NAG* N-acetyl-β-D-glucosaminidase, *BJP* Bence Jones protein

**Fig. 1:**
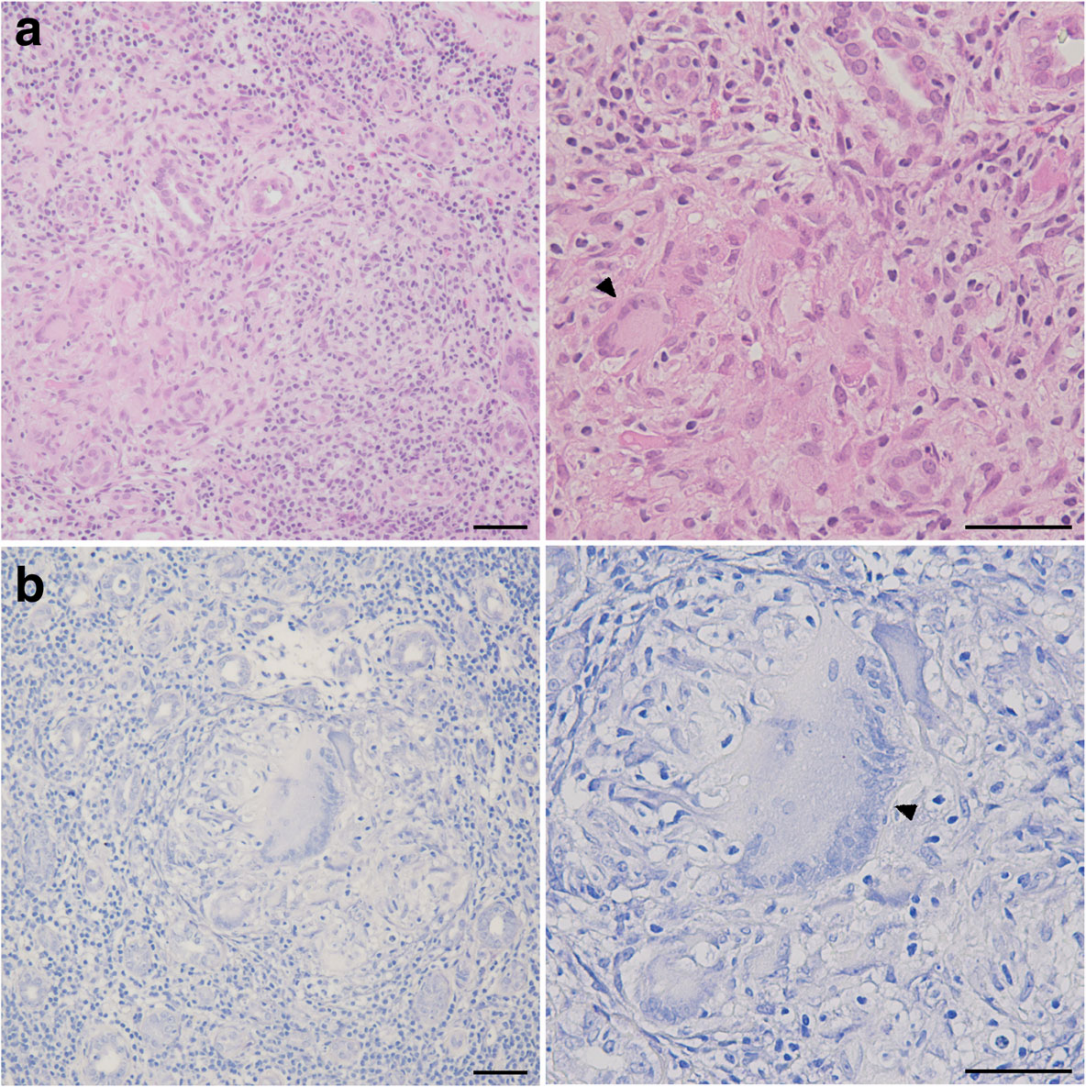
**a** Hematoxylin and eosin staining. Diffuse lymphocytic infiltration was observed in the tubulointerstitial area under low magnification. Under high magnification, non-necrotizing epithelioid cell granuloma with Langhans giant cells (arrowhead) was observed. **b** Ziehl-Neelsen staining. No acid-fast bacilli were observed under low magnification. The formation of a non-necrotizing epithelioid cell granuloma with Langhans giant cells (arrowhead) was observed under high magnification. Scale bars, 50 μm

**Fig. 2:**
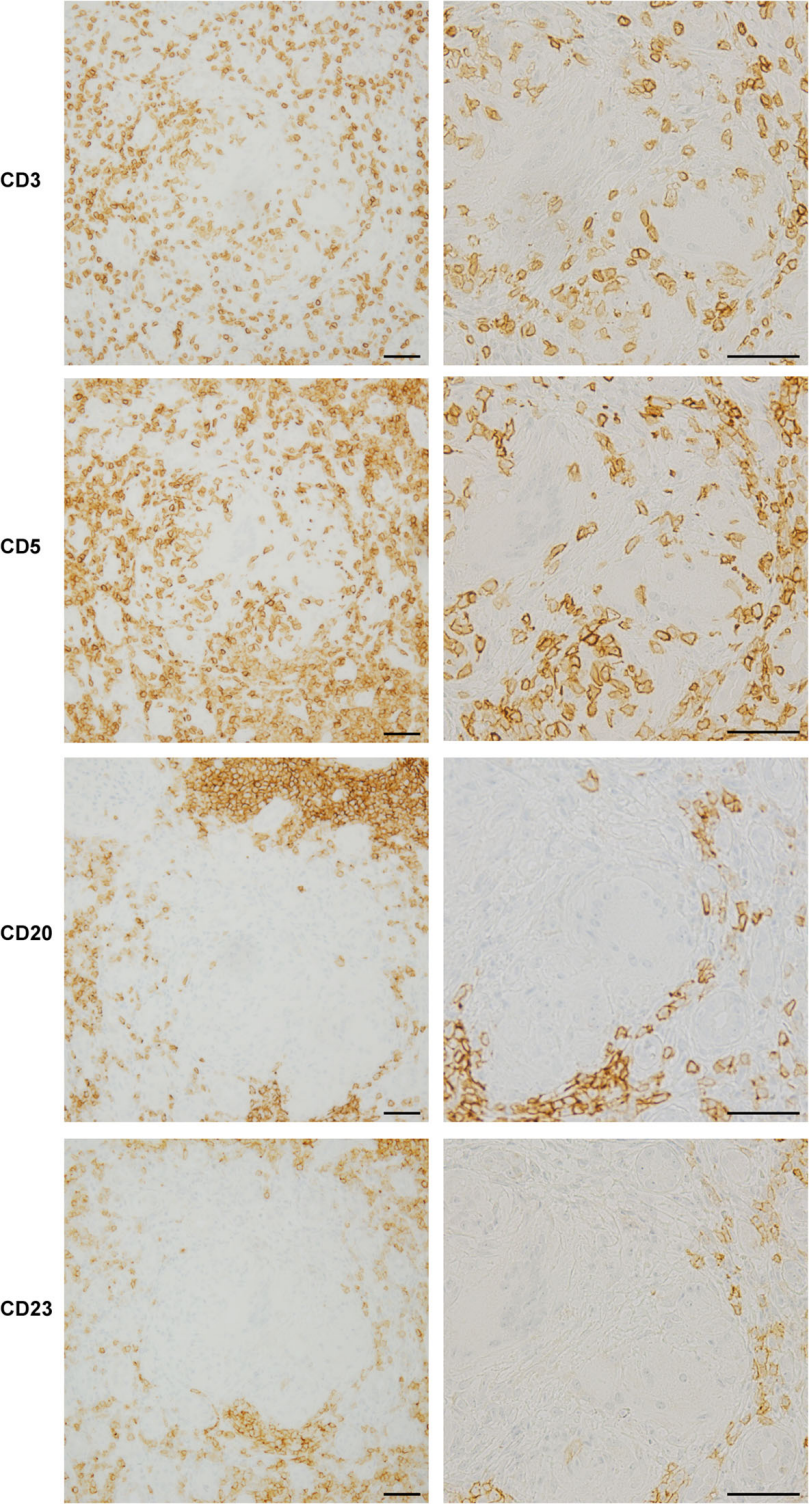
Immunohistochemical staining of CD3, CD5, CD20, and CD23. Under low magnification, the tubulointerstitial area was strongly positive for CD5 and CD20, positive for CD3, and weakly positive for CD23. Under high magnification, the inside or in the vicinity of the epithelioid cell granulomas was positive for CD3 and CD 5, and negative for CD20 and CD23. Scale bars, 50 μm

A renal biopsy obtained 53 glomeruli, 30 of which showed global sclerosis and 11 of which were collapsed without glomerular lesions. Immunofluorescence staining of immunoglobulin G (IgG), IgA, IgM, C3, C1q, and fibrinogen was negative. Tubulointerstitial injury with the interstitial infiltration of lymphocytes was observed in 90% of the total area, and there were some non-caseating epithelioid cell granulomas with Langhans giant cells (Fig. 1a). Ziehl-Neelsen staining for acid-fast bacilli was negative (Fig. 1b). Immunohistochemistry was performed with the T-cell marker CD3 and B-cell markers CD5, CD20, and CD23 (Fig. 2). The interstitial area was strongly positive for CD5 and CD20, positive for CD3 and weakly positive for CD23. The area inside or in the vicinity of the epithelioid cell granulomas was positive for CD3 and CD5 and negative for CD20 and CD23.

We determined that progressive renal dysfunction had occurred due to the diffuse infiltration of CLL cells in the interstitial area of the kidneys, and the patient was treated with two cycles of cyclophosphamide. The treatment was unable to attenuate the progression of the renal dysfunction, and the patient ultimately required maintenance hemodialysis due to end-stage renal failure.

## Discussion

We experienced a rare case of GIN due to the diffuse infiltration of CLL cells in a patient who developed progressive renal failure. The clinical course of the present case was quite similar to the first reported case of GIN due to the diffuse infiltration of CLL cells, which involved a patient who developed end-stage kidney failure over a four-year period [8]. CD3 and CD5 were detected in the interstitial area in both cases, and the renal function did not recover after the treatment of CLL. In both cases, the infiltration of CLL cells was diffuse. Another study reported the case of a patient with GIN with CLL who was negative for CD5 and who received hemodialysis after 18 months [9]. There was also a report of five cases of GIN secondary to CLL in which the renal dysfunction improved after corticosteroid treatment with or without CLL-directed chemotherapy in four of the five cases [10]. Among these four cases, the extent of CLL cell infiltration was mild to moderate, while the fifth case, in which the renal dysfunction did not improve, showed diffuse infiltration similarly to the present case and that of Kamat et al. [8].

The etiologies of renal failure in CLL patients include acute renal tubular necrosis, urate nephropathy, light chain deposition disease, obstructive nephropathy, amyloidosis, hypercalcemia, glomerulonephritis, cryoglobulinemia, tubulointerstitial nephritis, and drug-induced kidney injury [11]. Regarding kidney injury due to the direct infiltration of CLL cells, the invasion of CLL cells compresses the renal tubules secondarily, resulting in obstruction and ischemia in the kidney [12]. We observed similar findings in the present case. The renal biopsy showed that 57% (30 out of 53) of the glomeruli had global sclerosis, and 21% (11 out of 53) of those were collapsed. These lesions might occur as a consequence of arteriosclerotic changes. However, the sizes of the kidneys were within the normal limits, and neither intimal thickening of interlobular arteries nor hyalinosis of arterioles was observed in the renal biopsy. Therefore, these lesions were very likely to have been caused by compression of arterioles due to the diffuse infiltration of CLL cells. The patient’s renal function gradually decreased as the leukocytosis progressed, indicating that the CLL cells gradually compressed the arterioles and caused progressive renal dysfunction. The urinalysis showed no hematuria with mild proteinuria, and the markers for renal tubular injury were elevated, which was unusual for a pure interstitial nephritis and might represent a clinical feature specific to the ischemic changes induced by the diffuse infiltration of CLL cells. Compared with the two similar previous cases whose chronic renal function did not recover, there was no hematuria in the fifth case of Nasr et al. [10], while the hematuria data were not available in the case of Kamat et al. [8]. These findings suggested the absence of hematuria in cases wherein the progression of the diffuse infiltration of CLL cells occurred gradually.

In the present case, the pathological diagnosis was GIN due to the direct infiltration of CLL cells because CD5 and CD20 were positively detected in the lymphocytes in the tubulointerstitial area. There were no signs of generalized sarcoidosis because the patient had a normal serum ACE level with a normal chest radiograph and showed no uveitis. The formation of granuloma occurs concomitantly with infection, malignant tumors including lymphoma, and foreign bodies. The incidence of granuloma formation in patients with malignant neoplasms is reported to be 4.4% in patients with malignant tumors, 13.8% in those with Hodgkin’s lymphoma, and 7.3% in those with non-Hodgkin’s lymphoma [13, 14]. It is hypothesized that such granulomas form through the T-cell-mediated immunological response against tumor-cell-derived soluble antigens [14, 15].

Although the treatment of CLL can include combinations of corticosteroids, chlorambucil, vincristine, cyclophosphamide, rituximab, fludarabine, or radiation therapy, the responses to treatment vary; some patients show complete remission, while others are refractory to treatment [12]. Since our hematologist diagnosed the patient as Rai stage II, he chose to undergo treatment with cyclophosphamide instead of corticosteroids. The infiltration of CLL cells was so diffuse in the present case that we considered treatment with corticosteroids unlikely to be effective. Indeed, the two similar previous cases who had progressive renal dysfunction due to the diffuse infiltration of CLL cells needed maintenance hemodialysis despite being treated with corticosteroids and chlorambucil or rituximab [8, 10]. Unfortunately, the progressive renal failure in the present case did not improve with cyclophosphamide monotherapy. ACE inhibitors and angiotensin II receptor blockers were not tried because he was normotensive and the proteinuria was relatively mild. However, the use of an ACE inhibitor may have been an option for resolving the proteinuria in the present case, as a previous report assessed the timing and intensity of ACE inhibitors with regard to their antiproteinuric effect [16]. Renin inhibitors may be another therapeutic option for resolving proteinuria [17].

## Conclusion

In conclusion, we experienced a case of GIN due to the direct infiltration of CLL cells. When progressive renal dysfunction is observed in CLL patients, renal lesions of CLL cells should be considered.

## Abbreviations

ACE: Angiotensin-converting enzyme
CD: Cluster of differentiation
CLL: Chronic lymphocytic leukemia
GIN: Granulomatous interstitial nephritis
IgG: Immunoglobulin G
IGRA: Interferon gamma release assay
PET-CT: Positron emission tomography–computed tomography
